# Correction: Zhang, J.; Liu, K. Neural Information Squeezer for Causal Emergence. *Entropy* 2023, *25*, 26

**DOI:** 10.3390/e25101387

**Published:** 2023-09-28

**Authors:** Jiang Zhang, Kaiwei Liu

**Affiliations:** 1 School of Systems Sciences, Beijing Normal University, Beijing 100875, China; kevinliunxt@163.com; 2 Swarma Research, Beijing 100085, China

There was an error in the original publication. We found that due to previous negligence, some important content was missing in the previous manuscript [1]. It is a lemma in the appendix that supports the theorem.

A correction has been made to ***Appendix B***:

**Lemma** **A1.** *(Bijection mapping does not affect mutual information): For any given continuous random variables* X *and* Z*, if there is a bijection (one to one) mapping* f *and another random variable* Y *such that for any* $x\in Dom\;\left(X\right)$  *there is a* y=f (x)∈ Dom (Y)*, and vice versa, where* $Dom\;\left(X\right)$ *denotes the domain of the variable* X*, then the mutual information between* X *and* Z *is equal to the information between* Y *and* Z*, that is:*

(A15) $$I\;\left(X;Z\right)=I\;\left(Y;Z\right).$$

**Proof.**  Because there is a one to one mapping f:X→ Y, we have:

(A16) $$p_{X}\left(x\right)=p_{Y}\left(y\right)\left|det\;J\right|,$$

where $p_{Y}$ and $p_{X}$ are the density functions of X,Y, $J={\left.\frac{\partial f}{\partial X}\right|}_{x}$ is the Jacobian matrix of f, and if we insert Equation (A16) into the expression of the mutual information of I (X;Z), and replace the integration for x with the one for y, we have:

(A17) $$\begin{array}{cc} I\;\left(X;Z\right) & =\int_{X}\int_{Z}^{\;}p_{XZ}\left(x,z\right)\cdot ln\frac{p_{XZ}\left(x,z\right)}{p_{X}\left(x\right)p_{Z}\left(z\right)}\cdot dz\cdot dx\\ & =\int_{X}\int_{Z}^{\;}p_{X}\left(x\right)p_{Z|X}\left(z|x\right)\cdot ln\frac{p_{X}\left(x\right)p_{Z|X}\left(z|x\right)}{p_{X}\left(x\right)p_{Z}\left(z\right)}\cdot dz\cdot dx\\ & =\int_{Y}\int_{Z}^{\;}|det(J)|\cdot p_{Y}\left(y\right){\cdot p}_{Z|Y}\left(z|y\right)\cdot ln\frac{p_{Z|Y}\left(z|y\right)}{p_{Z}\left(z\right)}\cdot \left|det\left(J^{-1}\right)\right|\cdot dz\cdot dy\\ & =I\;\left(Y;Z\right). \end{array}$$

And Equation (A17) can also be proved because of the commutativeness of the mutual information. □

Due to the insertion of Lemma A1, the number of following Lemma are changed.

The authors state that the scientific conclusions are unaffected. This correction was approved by the Academic Editor. The original publication has also been updated.
